# Quality of life for older patients with cancer: a review of the evidence supporting melatonin use

**DOI:** 10.1007/s40520-020-01532-0

**Published:** 2020-03-31

**Authors:** Angeline Ginzac, Sophie Dubois, Marie-Odile Hager, Fabrice Kwiatkowski, Judith Passildas, Julian Biau, Catherine Abrial, Marie-Ange Mouret-Reynier, Emilie Thivat, Xavier Durando

**Affiliations:** 1 Université Clermont Auvergne, INSERM, U1240 Imagerie Moléculaire et Stratégies Théranostiques, Centre Jean PERRIN, 63011 Clermont-Ferrand, France; 2 Division de Recherche Clinique, Délégation Recherche Clinique et Innovation, Centre de Lutte contre le Cancer, Centre Jean PERRIN, 58, rue Montalembert, 63011 Clermont-Ferrand Cedex 1, France; 3 Centre d’Investigation Clinique, UMR501, 63011 Clermont-Ferrand, France; 4 Département d’Oncologie Médicale, Centre Jean PERRIN, 63011 Clermont-Ferrand, France; 5 CHU Clermont-Ferrand, Gérontopole, 63003 Clermont-Ferrand, France; 6 Département de Radiothérapie, Centre Jean PERRIN, 63011 Clermont-Ferrand, France

**Keywords:** Melatonin, Older patients, Cancer, Quality of life, Chemotherapy

## Abstract

**Purpose:**

The proportion of older populations living with cancer is on the increase. Maintaining or improving their quality of life (QoL) has become an important goal in the treatment of cancer and has become an endpoint in clinical trials. Melatonin regulates a wide variety of physiological functions and is involved in the initiation of sleep and the improvement of QoL. With age, the secretion of melatonin decreases and could lead to a deterioration in QoL.

**Methods:**

Literature searches were conducted using the PubMed database. The search terms and derivatives of “metastatic cancer”, “older patients”, “quality of life” and “melatonin” were used. Titles and abstracts were screened to identify whether studies were relevant for full-text screening.

**Results:**

There is major concern about the symptoms older cancer patients encounter during treatment because they can impact their QoL. Melatonin supplementation presents several benefits for older patients: improvement in survival, decrease in symptoms induced by cancer and cancer treatment, and also improvements in quality of life.

**Conclusion:**

It therefore seems appropriate to study the impact of melatonin supplementation during cytotoxic therapy on QoL among elderly patients with metastatic cancer. The use of melatonin as a therapeutic strategy seems particularly suitable for elderly patients, a population known to secrete significantly less melatonin. However, to date, no studies have been conducted in this population.

## Background

In 2018, 18.1 million new cancers were recorded worldwide and 9.6 million deaths were attributed to cancer [1]. Europe accounts for 9% of the world’s population, but for 23.4% of all cancer cases and for 20.3% of cancer deaths [1]. For most types of cancer, incidence rates increase with age. In the USA, an increase in cancer incidence from 2010 to 2030 of 67% has been estimated for people of 65 years or older, compared to an increase of only 11% among patients younger than 65 years [2]. The management of cancer among elderly patients is a public health problem.

The age of 70 is a threshold commonly used in clinical trials in oncology, but some authors use 65 as a reference age [3]. The European Medicines Agency (EMA) considers 65 years as a cut-off for the definition of old age (E7 Studies in Support of Special Populations: geriatrics). Despite the high frequency of cancer in the elderly population, these patients are under-represented in clinical trials [4–6]. Among studies by the National Cancer Institute on early stage breast cancer, only 18% of patients were over 65 years, while this population accounts for 49% of eligible patients [7]. Thus, we note here the importance of taking into account this population in clinical trials to improve current therapeutic treatment.Cancers among older patients are often diagnosed late and they often remain undertreated [8]. Late diagnoses can affect survival in this subpopulation [9]. The main reasons for the late diagnosis of cancer are comorbidities, the lack of a supportive social environment, difficulties in accessing public transport and deteriorating cognitive function [10]. A detailed study of the literature data has shown that compared to younger patients, older patients are subjected to a higher percentage of certain types of cancer at advanced stages [8], and also a higher percentage of non-staged cancers and a higher percentage of non-histological or non-cytological confirmations of cancer [11]. In fact, the study by De Rijke et al., performed on a cohort of 6911 patients aged 50 or over showed that, overall, 16% of patients were not treated, with the percentage rising to 22% for those over 70 years of age [11].

Elderly patients present particular characteristics that make the choice of the appropriate treatment more difficult to determine. The elderly population is very heterogeneous and cancer treatment for these patients requires knowledge not only of the disease but also of the physiological and pathophysiological features associated with aging. In oncology, as all anti-cancer drugs have side effects, it is important to consider the benefits and the risks associated with these treatments, especially in the elderly population. They are indeed more vulnerable to treatment toxicities and are more likely to have side effects [12]. Indeed, age is associated with several physiological changes in organ function that could alter drug pharmacokinetics and have an impact on cytotoxic chemotherapy tolerability and toxicity [12]. For example, we can mention the decline in renal function [13], diminishing bone marrow reserves [14], anaemia [15], poor nutrition [16] or even changes within the gastrointestinal system [17]. Geriatric syndromes (e.g., impaired vision and hearing, incontinence, poor nutrition) engender additional considerable difficulties for the treatment of the elderly [18].

In addition, comorbidities have an impact on the choice of treatment (e.g., anthracyclin, increasing the risk of cardio-toxicity such as heart failure, cisplatin, increasing the risk of renal failure) and they could lead to poly-medication, which contributes to increased chemotherapy toxicity among older patients [12].

As described above, the older cancer population is heterogeneous and complex (comorbidities, general health, drug interactions, dosage adjustment) and maintaining quality of life (QoL) is a major challenge in the care of these patients [19, 20]. In advanced disease, treatment is palliative, where the aim is to control the disease and pain, limit toxicities and maintain an overall level of QoL. However, with age, the pineal gland produces less melatonin. Melatonin regulates a wide variety of physiological functions and is involved in the initiation of sleep and the improvement of QoL. Thus melatonin has potential value in cancer pathologies in addition to the standard treatment to prevent and/or reduce the symptoms associated with cancer and its management, such as asthenia, depressive syndromes, sleep disorders, cognitive decline or even performance status, these dimensions being constitutive of QoL. The absence of any particular toxicity [21–23] in various clinical trials and the possible anti-tumor efficacy of melatonin strengthen its beneficial roles. Finally, the use of melatonin as a therapeutic strategy seems particularly suitable for older patients, a population known to secrete significantly less melatonin. However, to date no studies have been conducted in this population. This article aims to review the interest of melatonin for older patients with cancer to improve their quality of life during treatment.

## Methods

A literature search was conducted on PubMed database. The search terms and derivatives of “metastatic cancer”, “older patients”, “quality of life” and “melatonin” were used. Titles and abstracts were screened to identify whether studies were relevant for full-text screening. Only English-language studies were included. Articles were selected for full-text screening if the title or abstract mentioned one or more search terms.

### Older patients with cancer and quality of life

In 1993, the World Health Organization (WHO) defined QoL as “an individual’s perception of their position in life in the context of the culture and the value systems in which they live and in relation to their goals, expectations, standards and concerns” [24]. Health-related QoL (HRQoL) is a multidimensional concept including the physical, mental and social fields and the symptoms related to the disease and treatment [25]. Several studies have highlighted the prognostic value of data from HRQoL measures for cancer patients, especially in the advanced stages [26–29].

Two measurement tools are primarily used in oncology: the Functional Assessment of Cancer Therapy (FACT) [30] and the European Organization for Research and Treatment of Cancer Quality of Life Questionnaire Core 30 (EORTC QLQ-C30) [31], appropriate for self-evaluation and applicable across a wide range of settings. The core questionnaire is supplemented by disease-specific modules, e.g. breast, lung, head and neck cancer, palliative care, and a specific module for the elderly (QLQ ELD14).

There is major concern about the symptoms older cancer patients have to cope with during treatment because they can impact their QoL. Particularly at the metastatic stage, the aim of therapeutic management is to control the main symptoms. Thus QoL research incorporates these dimensions and is particularly relevant for decisional management for older patients with metastatic cancer. It is important to underline that the cancer treatment itself influences QoL (e.g., chemo-induced anaemia, asthenia, anorexia, reduced mobility, or even depressive symptoms) [32]. Pain, fatigue, insomnia and mood disturbance are the four most common symptoms and the most distressful that were reported by elderly patients with cancer during the illness and treatment [33]. The interrelations in this cluster of symptoms could affect QoL [34].

A study on 120 patients (> 65 years), receiving cancer therapy showed that the patients had numerous symptoms, with a mean number of 5 ± 3 symptoms per patient. Mood disturbance was the most prevalent (87%). The high-symptom group obtained a significantly lower mean Karnofsky performance score and FACT-G sub-scale and total scores (*p* < 0.01) (Cheng and Yeung, 2013). A recent study on 74 older patients with solid tumours showed a significant increase in excessive daytime sleepiness (EDS) after chemotherapy [from 6 patients (8.1%) exhibiting EDS at baseline to 16 patients (21.6%) after chemotherapy; *p* < 0.01]. The authors concluded that this impact of chemotherapy on daytime sleepiness could affect quality of life [35].

Furthermore, the management of cancer, and chemotherapy in particular, can induce a disruption of the circadian system, which can impact quality of life negatively. Mormont et al., in a study on 200 metastatic colorectal cancer patients, demonstrated a link between the rest-activity rhythm and QoL, showing a positive correlation between 24 h rhythm indicators and the global QLQ-C30 global score [36]. Innominato et al., in a study on 96 patients with metastatic colorectal cancer also showed that disruption of the circadian system was associated with lower QoL, global and in various domains, including the social environment [37]. The same team conducted another study on 237 metastatic colorectal cancer patients. They highlighted that fatigue and anorexia were more marked among patients with circadian disruption, and that QoL was better for patients with a robust circadian rhythm. The authors concluded that circadian disruption was associated with cancer symptoms and had an impact on physical and social functioning. Furthermore, they showed that the circadian system was important in the level of HRQoL for patients with cancer [38].

Several studies have shown a link between circadian rhythm disruption and survival among patients with cancer especially in metastatic colorectal cancer [39], advanced breast cancer [40], and lung cancer [41]. In the study by Innominato et al., circadian disruption among patients with metastatic colon cancer was a pejorative prognostic factor for overall survival, independently from other known prognostic factors [37]. The disruption of the circadian clock can also be caused by the chemotherapy itself. A study on 77 patients showed that chemotherapy-induced circadian disruption was also associated with significantly shorter survival [42]. Thus, these results justify the development of specific therapies aiming to restore the circadian system, which could potentially improve survival among cancer patients undergoing treatment.

### Interrelation between melatonin, older patients with cancer and quality of life

Melatonin is a compound synthesized by the pineal gland in the human brain. It is considered as a hormone regulating the circadian day–night rhythm. Tryptophan and serotonin are the precursors of melatonin. The regulation of melatonin synthesis is controlled by the light–dark cycle, acting through neural activation of the anterior hypothalamus CNS, via the axons of the retinal ganglion cells running from the optic nerves and forming the retino-hypothalamic tract). The secretion of melatonin has a typical circadian rhythm, reaching a peak value (80–150 pg/ml) between midnight and 3 a.m.[43, 44].

Since its discovery, many in vitro studies and animal models have been developed to determine the specific roles of melatonin in the body: antioxidant and onco-static properties [45, 46]; an anti-angiogenic role [47]; control of the immune system [48]; its role in sleep (reduced sleep latency and induction of sleepiness and drowsiness) [49]; its role in disorders of the biological rhythms [50].

Melatonin is metabolized in the liver and excreted in the urine as 6-sulfatoxymelatonin [51]. Due to the rapid absorption and short half-life of melatonin (40–50 min) after intake of an exogenous sustained-release formulation, a maximum plasma concentration occurring between 20 and 240 min is observed, followed by a decline after less than one and a half hours, depending on the dose [52]. Maintaining effective body concentrations of melatonin throughout the night thus requires either a high dose or an exogenous sustained-release formulation.

For example, Circadin^©^ is an exogenous sustained-release formulation of melatonin, which overcomes the rapid clearance of the hormone in the intestine over an extended period of time, thus mimicking the physiological pattern of melatonin secretion. Thereby, the peak plasma concentration is reached 2.6 h after intake and lasts an additional three and a half hours before falling, covering the whole night-time duration. In 2007, Circadin^®^ received its authorization on the European market as a monotherapy. It should be taken 2 h prior to bedtime and it can be used in the short-term treatment of primary insomnia among adults aged 55 and over. This authorization is based on results from two studies on patients (> 55 years) with primary insomnia [53, 54]. These studies and a meta-analysis [55] showed a significant benefit on sleep latency. This benefit is similar to that obtained from the already marketed hypnotics (sleep latency shortened by 25 min on average). In addition, these studies showed significant benefits in sleep quality, morning alertness and patient QoL. The aforementioned studies also showed that tolerance of the treatment is comparable to that of a placebo. After stopping the treatment for three nights, sleep quality deteriorates, but is still better among patients taking Circadin^®^, suggesting that there are not only no withdrawal symptoms and no risk in stopping treatment, but also that the treatment has a residual beneficial effect.

In these clinical trials, the most common side effects reported were headache, nasopharyngitis, back pain and arthralgia; they were observed in both the Circadin^©^ and the placebo groups.

With its role in the control of the circadian rhythm, we can hypothesize that, an intake of melatonin could have a beneficial effect on QoL and systemic symptoms. It can be remarked that, in humans, the pineal gland becomes less functional with age and melatonin levels gradually decline through life [56]. The causes of the decrease in melatonin secretion are not fully understood at this stage, and several hypotheses have been suggested, such as CNS degeneration, calcification of the pineal gland, or abnormal transmission of light signals [57]. Another hypothesis suggested is a decrease in melatonin receptors (MT1) in the brain, which is age related [58].

Between 40 and 70% of people suffer from sleep disorders, potentially affecting their psychological, social and cognitive functioning, and thus affecting their QoL [59]. Melatonin secretion is inversely correlated with sleep disturbances during the aging process. One study also demonstrated a link between reductions in urinary melatonin and poor quality of sleep in the elderly, and delayed sleep phase, identified by comparison with a control group of the same age [49].

### Benefits of melatonin supplementation for older cancer patients

Table 1 summarizes the main findings described in this section.

**Table 1:** Benefits of melatonin supplementation for cancer patients

References	Nb of patients	Endpoints	Design	Dosage MLT	Outcome	Level of evidence
Innominato [37]	32	To assess the effect of MLT on circadian biomarkers, sleep, and quality of life in metastatic breast cancer patients	Prospective open label, phase II trial	5 mg/day taken orally at each patient’s usual bedtime for 2 months	Daily bedtime MLT therapy is associated with reduction in sleep fragmentation, increases in sleep duration, improvements in self-rated sleep quality and improvement in global QoL and pertinent social and cognitive domains, as well as reduction in fatigue severity. Without any short-term toxicity	1c
Lissoni [64]	1140	Efficacy of MLT in patients with untreatable advanced solid tumors who had responded to previous standard anticancer therapies and for whom no other effective conventional treatment was available	Randomization according to tumor type: supportive care alone or supportive care + MLT	20 mg/day during the dark period	MLT may be effective in the prevention of both cancer progression-related symptoms and chemotherapy-induced toxicity: role in the supportive care of cancer patients MLT may have potential anticancer activity whether given alone or in association with cancer chemotherapy. The 1-year survival curve achieved in patients concomitantly treated with MLT was significantly higher than that for patients who received chemotherapy alone (*P* < 0.05)	1b
	200	efficacy of concomitant MLT administration on chemotherapy-induced toxicity and therapeutic activity in previously untreated patients with metastatic solid tumours who had chemo-resistant cancer and a good clinical status	Randomization according to the chemotherapeutic regimen: chemotherapy alone or chemotherapy + MLT	20 mg/day during the dark period		
Cerea [60]	30	To evaluate the influence of a concomitant administration of MLT on CPT-11 therapeutic activity in metastatic colorectal cancer patients progressing after at least one previous chemotherapeutic line containing 5 FU	Randomization: treatment with CPT11 alone or CPT11 + MLT (CPT11: I.V. 125 mg/m^2^/week for 9 consecutive week)	orally 20 mg/day during the dark period of the day	This preliminary study shows that the efficacy of weekly low-dose CPT-11 in pretreated metastatic colorectal cancer patients may be enhanced by a concomitant daily administration of the pineal hormone MLT, according to the results previously reported for other chemotherapeutic agents	1b
Lissoni [64]	40	MLT could amplify the efficacy of TMX in post-menopausal metastatic breast cancer patients with negative ER,	Randomized study TMX (tamoxifen) versus TMX + MLT	20 mg/day orally in the evening	No complete response was seen. Partial response rate was significantly higher in patients treated with TMX plus MLT than in those who received TMX alone The percent of survival at 1 year was significantly higher in patients treated with TMX + MLT than in those treated with TMX alone The association of the pineal hormone MLT may make TMX effective in ER-negative metastatic breast cancer patients	1b
Madsen [63]	48	To investigate whether administration of an oral dose of 6 mg MLT before bedtime perioperatively in breast cancer surgery could change sleep outcomes measured by actigraphy	Double blind, placebo-controlled, randomized clinical trial 27 patients received 6 mg MLT 21 patients received placebo	6 mg/day approximately 60 min before bedtime 3 nights preoperatively until at least 1 week postoperatively	Administration of 6 mg oral MLT significantly increased sleep efficiency and reduced waked after sleep onset for the entire 2-week postoperative period But MLT had no effects on other objective sleep outcomes or on subjective sleep quality	1b
Hansen [65]	54	Investigate whether MLT could lower the risk of depressive symptoms in women with breast cancer in a three-month period after surgery and assessed the effect of melatonin on subjective parameters (anxiety, sleep, general well-being, fatigue, pain and sleepiness)	Randomized, double-blind, placebo-controlled trial 28 patients received MLT 26 patients received placebo	6 mg/day 1 h before bedtime for 1 week preoperatively and 12 weeks ostoperatively	MLT significantly reduced the risk of depressive symptoms in women with breast cancer during a three-month period after surgery	1b
Del Fabbro [67]	48	To compare MLT with placebo for appetite improvement in patients with cancer cachexia (patients with advanced lung or GI)	Randomized, double-blind, 28-day trial of MLT 20 mg at night versus placebo	20 mg/day at night during 28 days	No significant difference between groups for appetite or other symptoms, weight, FAACT score, toxicity, or survival baseline to day 28 In cachectic patients with advanced cancer, oral MLT 20 mg at night did not improve appetite, weight, or quality of life compared with placebo The trial was stopped after interim analysis	1b
Sookprasert [68]	151 patients with histologically advanced NSCLC	Health-related QoL assessed with the Functional Assessment of Cancer Therapy—Lung	Randomized, double-blind, placebo-controlled trial A: 10 mg MLT B: 20 mg MLT C: Placebo	after 21 h on the first day of chemotherapy treatment and continue for 6 months	When used in combination with chemotherapy, MLT did not significantly affect survival and adverse events of patients with advanced NSCLC, but there was a trend for better HRQoL in the melatonin-treated groups, with a significantly better score in social well-being	1b
Wang [23]	761	To observe MLT effect on tumor remission, 1-year survival and side effects due to radiochemotherapy	Meta-analyses of eight randomized clinical trials (patients with solid tumor cancers)	20 mg orally, once a day	MLT significantly improved: the complete and partial remission (16.5 vs. 32.6%; RR = 1.95, 95% CI 1.49–2.54; *p* < 0.00001) 1-year survival rate (28.4 vs. 52.2%; RR = 1.90; 95% CI 1.28–2.83; *p* = 0.001) decreased radiochemotherapy-related side effects including thrombocytopenia (19.7 vs. 2.2%; RR = 0.13; 95% CI, 0.06–0.28; *p* < 0.00001), neurotoxicity (15.2 vs. 2.5%; RR = 0.19; 95% CI, 0.09–0.40; *p* < 0.0001) and fatigue (49.1 vs. 17.2%; RR = 0.37; 95% CI 0.28–0.48; *p* < 0.00001)	1a
Seely [22]	UK	1-year mortality, complete response, partial response, stable disease	Meta-analyses of 21 clinical trials (solid tumors)	UK	MLT may benefit cancer patients who are also receiving chemotherapy, radiotherapy, supportive therapy, or palliative therapy by improving survival and ameliorating the side effects of chemotherapy	1a

1a: systematic review or meta-analysis of randomized controlled trials (RCT) of good methodological quality and with homogeneity; 1b: individual RCT with narrow CI; 1c: non-controlled studies

*MLT* melatonin, *UK* unknown

#### Combined effects of melatonin with chemotherapy: improving survival

The effect of melatonin coupled with chemotherapy has been analysed in different cancers. Several trials support the hypothesis that melatonin enhances the effect of chemotherapy, especially in colorectal carcinoma Cerea et al. evaluated the effect of simultaneous administration of melatonin and camptothecin (Irinotecan^©^) to 30 patients with metastatic colorectal carcinoma. They showed that melatonin with Irinotecan^©^ was more effective in controlling the disease than Irinotecan^©^ alone [60].

A recent meta-analysis [23] of eight clinical trials tested the effect of melatonin supplementation among 761 patients treated with chemotherapy or radiation therapy for solid tumors in metastatic setting. The tested melatonin dose was 20 mg/day, prescribed concurrently with chemotherapy and/or radiotherapy. Melatonin significantly improved complete and partial remission rates (16.5 vs. 32.6%; risk ratio RR = 1.95, 95% CI 1.49–2.54; *p* < 0.00001) as well as 1-year survival (28.4 vs. 52.2%; RR = 1.90; 95% CI 1.28–2.83; *p* = 0.001). However, it is important to remain cautious because this meta-analysis included six studies conducted in the same centre on a small study population. Hence, an analysis using double-blind placebo and multicentre studies with larger patient samples is needed to evaluate the efficacy of melatonin in the treatment of cancer.

Another meta-analysis [22] included a larger group of trials (21 trials). The trials combined adjuvant chemotherapy with melatonin. Melatonin decreased 1-year mortality (RR = 0.60, 95% CI 0.54–0.67) and contributed to improved results for complete response, partial response and stable disease, with RR of 2.53 (1.36–4.71), 1.70 (1.37–2:12), and 1.15 (1.00–1.33), respectively.

#### Improvement of symptoms induced by cancer and its treatment

A study [21] on 200 patients with chemotherapy-resistant metastatic cancer, comparing chemotherapy versus chemotherapy + melatonin at a dose of 20 mg/day for at least 2 months, showed a significant reduction in chemotherapy-induced toxicities such as asthenia, thrombocytopenia, neurotoxicity and stomatitis. Another study conducted by the same team [21] showed a significant reduction in certain cancer-induced symptoms by providing melatonin to 1440 patients: 718 patients treated with supportive care alone, and 722 treated with supportive care + melatonin at 20 mg/day. The symptoms that improved were cachexia, asthenia, anorexia, depressive syndromes and thrombocytopenia.

A recent study conducted on 32 metastatic breast cancer patients who took 5 mg/day of melatonin for two months at bedtime showed a reduction in sleep fragmentation (*p* = 0,0015) and an increase in sleep duration [61]. Similarly, a double-blind, placebo-controlled, randomized study among breast cancer patients comparing 6 mg/day of melatonin (*n* = 27) to placebo (*n* = 21), from three nights before surgery until one week post-surgery, showed an increase in sleep efficacy in the melatonin group (*p* = 0,007) and a reduction in waking-after-sleep onset [62]. Also according to the above-mentioned meta-analysis [22, 23], melatonin significantly reduces symptoms such as asthenia, leukopenia, nausea and vomiting, hypotension, and thrombocytopenia. As most of these symptoms are included in QoL measures, their improvement by melatonin could translate into patient QoL improvement.

#### Role on quality of life

In a randomized study [63], an improvement of anxiety and depressive symptoms was observed among patients treated for metastatic breast cancer with Tamoxifen^©^ + melatonin. Another more recent study [64] showed a significantly lower risk of developing depressive syndrome after surgery for breast cancer among 54 patients receiving melatonin supplementation (6 mg/day, 3 months) versus placebo, RR 0.25 [95% CI 0.077–0.80].

An improvement of performance status (PS) was observed among patients with non-small cell lung cancer resistant to first-line chemotherapy with cisplatin, treated with supportive care + melatonin at 10 mg/day compared to patients treated with supportive care alone [65]. In addition, it is important to highlight that in many studies PS was correlated with QoL.

A double-blind trial [66] recently studied the effect of melatonin supplementation at 20 mg/day for 4 weeks on appetite in cachexia patients with advanced cancer. At the interim analysis of 48 patients, the trial was stopped early for futility, noting the absence of any advantage for the melatonin arm. In this study, QoL was also assessed, but no apparent difference between the two groups was observed. However, it is difficult to take these results into account, because the study was not designed to conclude on QoL, which was a secondary objective.

Only one double-blind trial [67], recently studied the effect of melatonin supplementation (10 or 20 mg/day) on QoL and reported a better meanadjusted QoL score (FACT-L) with a slightly significant better score (2.69 points, CI 0.01–5.38, *p* = 0.049) found for social wellbeing.

However, melatonin supplementation for older patients could entail a weakness. Indeed, this subpopulation, because of their numerous comorbidities, often has a lot of tablets to take; this would add yet one more tablet, which could be a limitation. It is also important to emphasize that sustained-release formulation of melatonin is not reimbursed by social security system in France (except for children with specific syndromes). So another weakness is the price of the supplementation: approximately 30 € for 30 tablets of Circadin^®^ 2 mg. Furthermore, in the studies, the dosage of melatonin varies from 5 mg/day to 20 mg/day while standard dosage of sustained-release formulation is 2 mg/day (Table 1). The smaller dosages lead to good results with an improvement in quality of sleep and depressive symptoms [61, 62, 64]. A systematic review, conducted on 16 articles, concluded that optimal dosage for melatonin supplementation in older adults is the lowest possible dose because it is closest to physiological circadian rhythm of melatonin [68].

## Conclusion

The use of melatonin as a therapeutic strategy seems particularly suitable for elderly patients, a population known to secrete significantly less melatonin. However, to date no studies have been conducted in this population. The elderly population is heterogeneous and complex (comorbidities, general health, drug interactions, dosage adjustment), and maintaining QoL is a major challenge in the care of elderly patients. In advanced disease, treatment is palliative and the aim is to control the disease and pain, limit toxicities and maintain an overall level of QoL. Thus, it seems appropriate to study the impact of melatonin supplementation during cytotoxic therapy on QoL among elderly metastatic cancer patients.
